# Loneliness as a mediation from social support leading to a decrease of health-related quality of life among PLWHIV

**DOI:** 10.3389/fpubh.2022.1067870

**Published:** 2023-01-04

**Authors:** Zhe Qian, Bing Li, Leyi Liao, Guichan Liao, Hongjie Chen, Juanqing Han, Tao Yu, Xuwen Xu, Jie Peng, Shaohang Cai

**Affiliations:** ^1^Department of Infectious Diseases, Nanfang Hospital, Southern Medical University, Guangzhou, China; ^2^Division of Hepatobiliopancreatic Surgery, Department of General Surgery, Nanfang Hospital, Southern Medical University, Guangzhou, Guangdong, China; ^3^Jinzhou Medical University, Jinzhou, China

**Keywords:** people living with HIV, loneliness, mediation, mental health, health related quality of life, social support

## Abstract

This study focused on the mental health of people living with HIV(PLWHIV) and explored their relationship between loneliness and perceived social support, health related quality of life (HRQoL) with a method of structural equation model. We collected clinical and psychological data from consecutively enrolled PLWHIV. A total of 201 PLWHIVs were enrolled and measured with self-reporting survey instruments of UCLA Loneliness Scale, Self-Rating Depression Scale, Self-Rating Anxiety Scale, Social Support Ratio Scale and Short Form Health Survey-36. The levels of loneliness, depression, anxiety, perceived social support and HRQoL were assessed. PLWHIV enrolled were divided into two groups of loneliness and non-loneliness based on their UCLA Loneliness Scale scores. Multivariable analysis indicated that being married is a protective factor associated with loneliness (OR = 0.226; *P* = 0.032). We further found the loneliness group had a higher level of depression (*P* < 0.001) and anxiety (*P* < 0.001), but lower level of HRQoL (*P* < 0.001) than the non-loneliness group. We found there was a positive linear correlation between social support and HRQoL among the enrolled PLWHIVs (r^2^ = 0.0592; *P* = 0.0005). A structural equation model (SEM) was established to evaluate whether the loneliness played as a mediation role between social support and HRQoL. The model showed loneliness as a mediation from social support leading to a decrease of HRQoL. Our findings showed a potential psychological pathway from social support to HRQoL, suggesting the need for interventions focusing on social support may improve poor HRQoL lead by loneliness.

## Introduction

With the widespread use of effective antiretroviral therapy (ART), great progress has been made in increasing the life expectancy of people living with HIV (PLWHIV) (1). HIV infection has been gradually developing into a chronic disease while the mental health of PLWHIV has become a focused issue, which was reported as a key factor affecting HIV treatment outcomes in high-income countries (2). However, the mental health of PLWHIV has only recently received the attention it deserves in low- and middle-income countries (3). PLWHIV are at increased risk of developing mental health conditions that range from acute stress reactions to neurocognitive disorders and negative emotional experiences (4). These negative conditions could undermine health-seeking behaviors and reduce adherence to treatment (5) which contributed to worse health-related quality of life (HRQoL) and lead to higher rates of mortality (6–8). The HRQoL of PLWHIV was usually evaluated with tools of MOS (Medical Outcome Study)-HIV and Functional Assessment of HIV Infection (FAHI) (9). HRQoL refers to patient reports of functioning and well-being in physical, mental, and social domains of life. But the terms quality of life and HRQoL have often been used interchangeably (10). The behaviors can be characterized by medical coping modes which have been proven as essential indicators for the improvement of QoL (11). While social support has been proved effectively protecting both physical and mental health (12). This relative relationship does not change according to gender (13). A previous study also indicated that the odds of participating in HIV risk behaviors decreased with social support (14).

However, the specific mechanism of how perceived social support leading to disorientating HRQoL still remains unknown. Recent studies (15, 16) indicated that loneliness may be an affecting factor for poor HRQoL. Similar findings were confirmed in pregnant mothers from Canada (17). Furthermore, loneliness can be a risk factor but also a mediator in complex psychosocial interaction networks (18). Loneliness is regarded as an important section of mental health defined as a subjective emotional experience different from the definition of social isolation referring to the actual number of social contacts a person has (19). A definition of loneliness is that it is a vital negative emotion defined as an unpleasant, subjective experience resulting from the lack or quality of social relationships (20). Loneliness is also established with increased psychiatric symptoms such as depression (21), anxiety (22, 23) and suicide attempts (24). Moreover, loneliness can have detrimental effects on physical health, e.g., coronary heart disease and stroke (25). All these risks that loneliness can bring make its early identification and prevention a vital concern, especially among vulnerable populations such as PLWHIV during the global pandemic of COVID-19 when the prevalence of loneliness increased than before (26). In order to improve intervention strategies to impose social support, and further alleviate depression and anxiety which may be associated with loneliness confirmed by former studies, and eventually, related downstream health consequences, research to disentangle mechanistic contributors is in great need.

Therefore, our study aimed to investigate the potential mediation mechanism of loneliness between social support and HRQoL in PLWHIV. The risk factors associated with loneliness in PLWHIV and the relationship between loneliness and depression, anxiety, HRQoL were also evaluated. The results of this study will help to understand the role that loneliness played in mental health perspectives and reveal a potential intervention target to improve HRQoL of PLWHIV.

## Methods

### Study population and design

Participants were recruited from a cohort regularly followed up in Nanfang Hospital, Southern Medical University with a method of randomized sampling. Eligible patients were (1) adults (≥18 years of age), (2) those confirmed to have HIV infection, and (3) had no underlying medical conditions that can interfere with comprehension of questionnaire content. Signed informed consent was obtained from all patients enrolled. All the patients completed the questionnaire surveys in a quiet room without any interference or disruptions. The institutional review board of the Nanfang Hospital has approved the study. A total of 201 patients were enrolled in our study and divided into loneliness and non-loneliness groups based on a UCLA Loneliness Scale cutoff value of 44 points (27). There were 94 patients in the loneliness group and 107 patients in the non-loneliness group. Demographic and clinical data are shown in Table 1.

**Table 1:** Baseline characteristics of loneliness group and non-loneliness group.

	**Loneliness group**	**Non-loneliness group**	***P*-value**
Age (years)	28.17 ± 7.79	27.96 ± 5.92	0.618
Gender			0.344
Male	89	105	
Female	5	2	
CD4 counts	249.25 ± 167.96	274.06 ± 191.72	0.270
CD8 counts	1063.60 ± 765.06	908.59 ± 584.62	0.103
HIV RNA viral load	5.36 ± 6.11	4.83 ± 5.24	0.069
AIDS stages			0.936
Yes	25	29	
No	69	78	
BMI	22.17 ± 18.81	20.69 ± 3.05	0.554
Marriage			0.020
Married	7 (7.69)	20 (19.23)	
Unmarried	84 (92.31)	84 (80.77)	
Education			0.579
Primary	52 (55.32)	55 (51.40)	
Advanced	42 (44.68)	52 (48.60)	
Income			0.471
Low-income	87 (88.42)	94 (92.47)	
High-income	7 (11.58)	11 (7.53)	
Regular fasting			0.550
Yes	24 (25.81)	21 (19.63)	
No	69 (74.19)	86 (80.37)	
Short sleep duration			0.348
Yes	89 (94.68)	98 (91.59)	
No	5 (5.32)	9 (8.41)	
Fitness			0.147
Yes	31 (32.98)	46 (42.99)	
No	63 (67.02)	61 (57.01)	
Alcohol consumption			0.895
Yes	26 (28.26)	30 (29.13)	
No	66 (71.74)	73 (70.87)	
Binge drinking			0.204
Yes	12 (12.90)	8 (7.48)	
No	81 (87.10)	99 (92.52)	
SF-36 scores	616.81 ± 129.22	729.77 ± 81.80	< 0.001

### Laboratory tests and demographic parameters

CD4+/CD8+ T lymphocyte counts were determined with a flow cytometer. HIV RNA was detected by polymerase chain reaction. Demographic and epidemiological information including age, gender, marriage, education, income, fasting, short sleep duration, fitness, alcohol consumption and binge drinking were also collected. Education level was defined as follows: primary, if patients received < 9 years of education, and senior, if patients received more than 9 years of education. Income level was defined as follows: high, if the income was more than 240,000 RMB per year, and low, if the income was < 240,000 RMB per year.

### Psychological measurements

#### Loneliness

Loneliness was measured with the UCLA Loneliness Scale Version 3, which consists of 20 questions with answer choices of “Never” (1 point) to “Often” (4 points), with total scores ranging from 20 to 80 (28). The higher the score, the higher the loneliness level. In our study, we considered 44 points as a cutoff value to divide the patients into a loneliness group and a non-loneliness group (27).

#### Depression

Depression was measured with the Self-Rating Depression Scale (SDS), which was designed by Duke University psychiatrist William W. K. Zung, MD, to assess the level of depression in patients diagnosed with depressive disorder (29). This questionnaire consists of 20 items scored using a 4-point scale, with the main item being the frequency of the defined symptom, based on the following criteria: “1” for no or little time; “2” for a small amount of time; “3” for a considerable amount of time; and “4” for most or all the time. All the scores are summed to determine the total score, which ranges from 20 to 80, and a score under 44 is considered normal without depression. A score of 45–59 is considered mild depression, 60–69 is considered moderate depression, and 70 or more is considered severe depression.

#### Anxiety

Anxiety was measured with the Self-Rating Anxiety Scale (SAS) (30). The SAS, developed by Zung in 1971, is like the Self-Rating Depression Scale (SDS) in terms of its construction and the way it is rated. The SAS consists of 20 items scored on a 4-point scale, which is the same as the SDS. All the scores are summed to determine the total score. The higher the score is, the more pronounced the tendency to experience anxiety. A score of 50–59 is considered mild anxiety, 60–69 is considered moderate anxiety, and 70 or more is considered severe anxiety.

#### Social support

Social support was assessed with the Social Support Ratio Scale (SSRS), which was designed by Xiao (31). Social support is divided into 3 categories in this scale: objective support, which is visible or tangible and includes direct material assistance, and the presence and participation of group relationships. The second category is subjective support, which is experienced or emotionally felt by an individual and refers to the emotional experience and satisfaction of being respected, supported, and understood in society and is closely related to an individual's subjective feelings. The final category is the utilization of social support. This scale has 10 items that are summed to determine the final score, which ranges from 0 to 40. The higher the score, the higher the degree of social support an individual receives. A score < 20 indicates that a subject receives only low levels of social support, 20–30 indicates general social support and 30–40 indicates satisfactory social support.

#### Health-related quality of life

Health-related quality of life (HRQoL) was assessed using the MOS (Medical Outcomes Study) short form health survey (SF-36) (32). The SF-36 is a 36-item, patient-reported survey of patient health. The original SF-36 stemmed from the MOS, which was conducted by the RAND Corporation. It is widely used in the areas of the quality of survival measurements in the general population, and the evaluation of the effectiveness of clinical trials and health policy assessments. As a concise health questionnaire, the SF-36 provides a comprehensive overview of the quality of patient survival in the following nine areas: (1) Physical Function (PF); (2) Role Physical (RP); (3) Bodily Pain (BP); (4) General Health (GH); (5) Vitality (VT); (6) Social Functioning (SF); (7) Role Emotional (RE); (8) Mental Health (MH); and 9 Reported Health Transition (HT). The scores from each area are summed to determine the total score. High scores represent good quality of life. These eight areas can be further divided into two main categories: (1) Physical Component Summary (PCS), including PF, RP, BP, and GH and (2) Mental Component Summary (MCS), including VT, SF, RE, and MH.

### Statistical analysis

In our study, we used the mean ± standard deviation, and categorical variables were used to express variables when appropriate. The chi-square test and *t*-test were used to determine whether the results were significantly different. Univariate and multivariate logistic regression analysis to determine factors related to loneliness among the PLWHIV. Simple linear regression was also used to determine the relationship between UCLA loneliness scale scores and SAS scores, SDS scores, and SF-36 scores which was performed by Pearson's correlation. The significance level was set as P < 0.05 (two-tailed). Data analysis and quality control procedures were performed using SPSS 26.0 (Chicago, USA). To further determine the latent mechanism of loneliness in the association between social support and HRQoL, we performed a path analysis with AMOS 25.0 version module (IBM). This was presented by a structural equation model (**Figure 3**). Standardized regression weights (β coefficients) were reported with their *P*-values. CMIN (chi-squared test), CMIN/DF, GFI (Goodness of Fit Index), NFI (Normed Fit Index), IFI (Incremental fit index), TLI (Tucker-Lewis's index), CFI (Comparative fit index) and RMSEA (Root Mean Squared Error of Approximation) were used to assess the model fit. CMIN/DF between 1 and 3, GFI, NFI, IFI, TLI, and CFI of > 0.95, and RMSEA < 0.05 indicate good model fit. HOELTER > 200 indicated an adequate sample size.

## Results

### Demographic data of the enrolled patients

A total of 201 patients were enrolled in this study, 94 of whom were determined to have loneliness and 107 of whom were not. The demographic and clinical characteristics are shown in Table 1. A higher proportion of PLWHIV being married was observed in the non-loneliness group (*P* = 0.020).

### Factors related to loneliness in PLWHIV

To determine the related factors associated with loneliness among PLWHIV, we conducted univariate and multivariate analyses. Results from the univariate analysis revealed that being married was an associated factor (OR = 0.350, 95% CI: 0.141–0.872, *P* = 0.024). However, in the multivariate analysis, we found that being married (OR = 0.226, 95% CI = 0.058–0.879, *P* = 0.0 32) and regularly fasting (OR = 2.524, 95% CI = 0.994–6.409, *P* = 0.051) were independent factors related to loneliness (Table 2).

**Table 2:** Factors associated with of loneliness among people living with HIV.

	**Univariate analysis**			**Multivariate analysis**		
	**OR**	**95%CI**	***P*-value**	**OR**	**95%CI**	***P*-value**
Age	0.991	0.955–1.028	0.617			
Gender	0.342	0.065–1.808	0.207			
CD4 counts	0.999	0.997–1.001	0.390			
CD8 counts	1.000	1.000–1.001	0.156			
HIV RNA	1.000	1.000–1.000	0.487			
Marriage	0.350	0.141–0.872	0.024	0.226	0.058–0.879	0.032
Education	0.854	0.490–1.490	0.579			
Income	0.696	0.258–1.875	0.473			
Fasting	1.484	0.766–2.874	0.242	2.524	0.994–6.409	0.051
Short sleep duration	1.386	0.767–2.504	0.279			
Fitness	0.653	0.367–1.160	0.146			
Alcohol consumption	0.959	0.515–1.785	0.894			
Binge drinking	1.833	0.715–4.701	0.207			

### Depression and anxiety levels among PLWHIV

**Figure 1:**
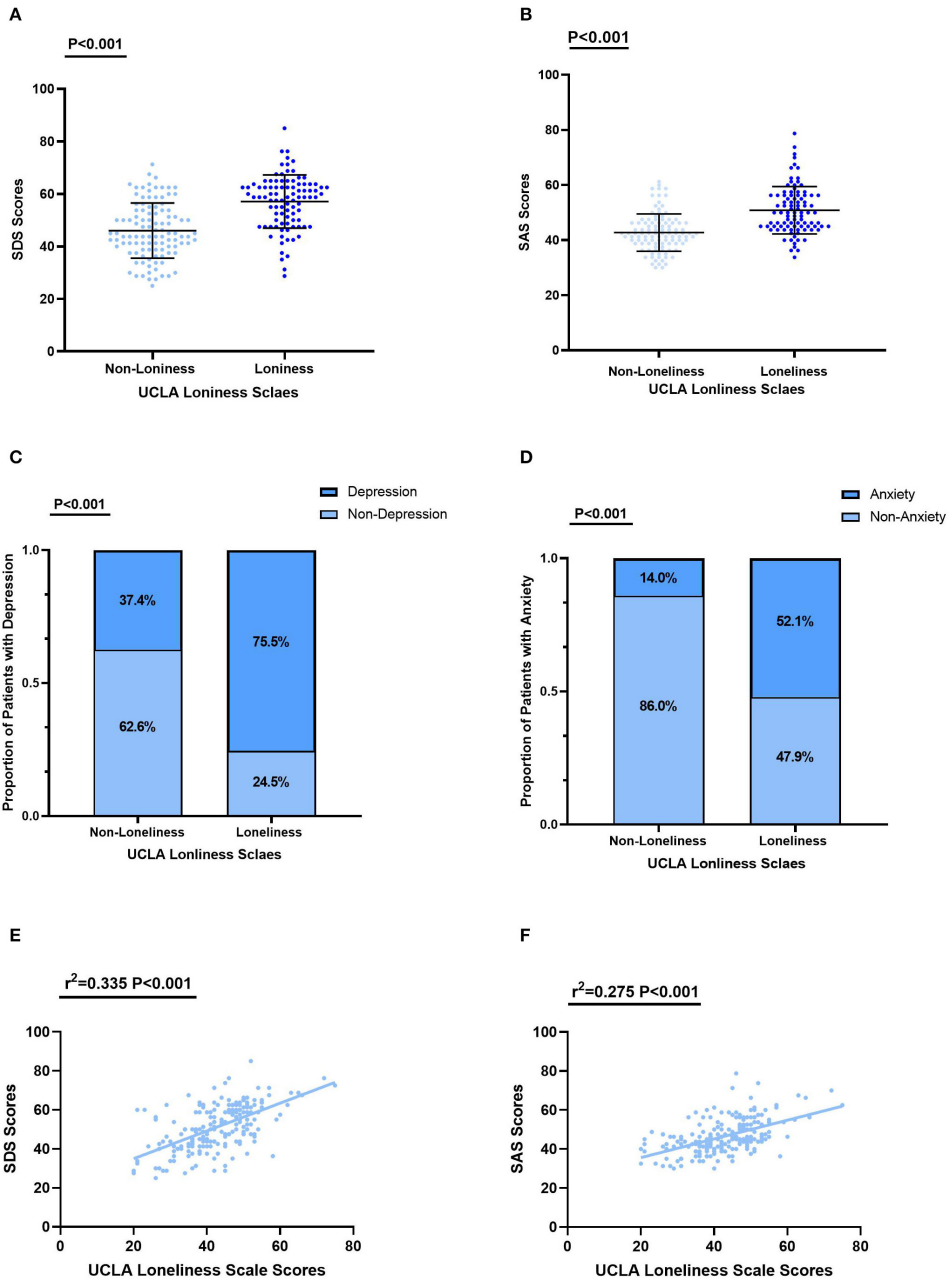
Levels of depression and anxiety in the loneliness and non–loneliness groups. **(A)** The depression score of PLWHIV with loneliness were 57.10 ± 10.12, significantly higher than in that without loneliness 46.02 ± 10.50 (*P* < 0.001). **(B)** The depression score of PLWHIV with loneliness were 52.85 ± 8.61, significantly higher than in that without loneliness 42.75 ± 6.79 (*P* < 0.001). **(C)** The proportion of diagnosed with depression was significantly higher in PLWHIV with loneliness than that without loneliness. **(D)** The proportion of diagnosed with anxiety was significantly higher in PLWHIV with loneliness than that without loneliness. **(E)** The relationships between SDS Scores and UCLA Loneliness Scale Scores. **(F)** The relationships between SDS Scores and UCLA Loneliness Scale Scores. SAS, Self–Rating Anxiety Scale; SDS, Self–Rating Depression Scale; UCLA, The University of California, Los Angeles.

The depression levels were higher in the loneliness group than the others (57.10 ± 10.12 vs. 46.02 ± 10.50, *P* < 0.001), as shown in Figure 1A. Similar trends were found in anxiety levels in PLWHIV enrolled (52.85 ± 8.61 vs. 42.75 ± 6.79, *P* < 0.001) (Figure 1B). The proportion of patients with depression in the loneliness group was 75.5% compared with 37.4% (*P* < 0.001) in the non-loneliness group (Figure 1C), while the proportion of anxiety in the loneliness group was 52.1% compared with 14.0% (*P* < 0.001) in the non-loneliness group (Figure 1D). Furthermore, we observed that PLWHIV with loneliness had higher rates of depression and anxiety of all levels than PLWHIV without loneliness (Supplementary Tables S1, S2).

To further evaluated the relationship between loneliness and anxiety or depression in PLWHIV, we conducted correlation analysis. Levels of depression (r^2^ = 0.335, *P* < 0.001) and anxiety (r^2^ = 0.275, *P* < 0.001) were significantly positively associated with the level of loneliness, as shown in Figures 1E, F.

### Association between social support and HRQoL

The association of perceived social support along with its three dimensions and HRQoL along with its two dimensions were assessed.

**Figure 2:**
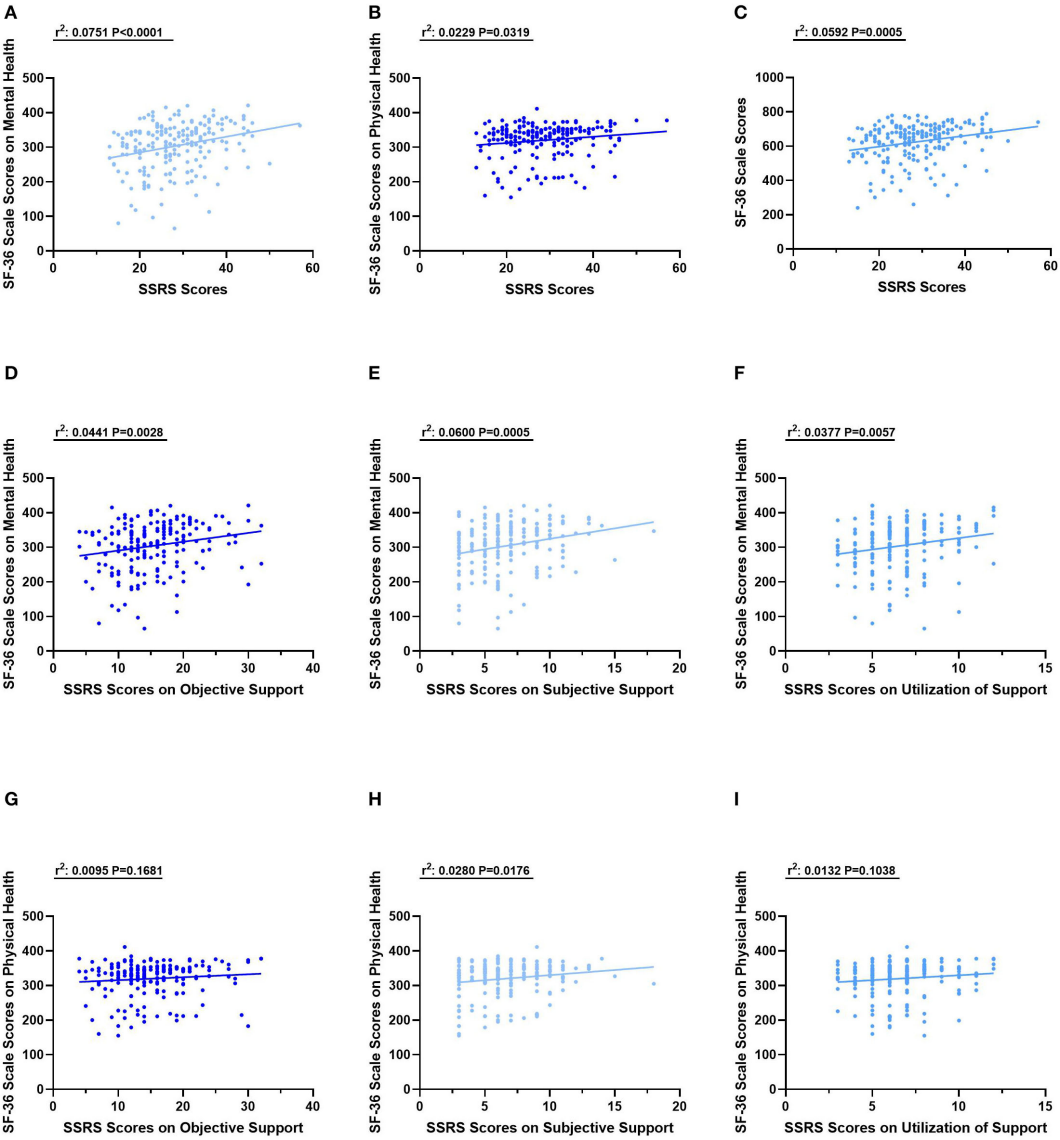
The relationships between SSRS scores and SF−36 scores. **(A)** The relationship between SSRS Scores and SF−36 Scores on Mental health. **(B)** The relationship between SSRS Scores and SF−36 scores on Physical health. **(C)** The relationship between SSRS Scores and total SF−36 scores. **(D)** The relationship between SSRS Scores on Objective Support and SF−36 scores on Mental health. **(E)** The relationship between SSRS Scores on Subjective Support and SF−36 scores on Mental health. **(F)** The relationship between SSRS Scores on Utilization of Support and SF−36 scores on Mental health. **(G)** The relationship between SSRS Scores on Objective Support and SF−36 scores on Physical health. **(H)** The relationship between SSRS Scores on Subjective Support and SF−36 scores on Physical health. **(I)** The relationship between SSRS Scores on Utilization of Support and SF−36 scores on Physical health.

The health-related quality of life of PLWHIV was positively correlated with perceived social support (r^2^ = 0.0592, *P* = 0.0005) and the similar relationship between its two dimensions of mental health (r^2^ = 0.0751, *P* < 0.0001) and physical health (r^2^ = 0.0229, *P* = 0.0319) were also determined (Figures 2A–C).

The relation between three dimensions of SSRS scores and SF-36 scale scores on mental health was also assessed. The SSRS scores on objective support (r^2^ = 0.0441, *P* = 0.0028), subjective support (r^2^ = 0.0600, *P* = 0.0005) and utilization of support (r^2^ = 0.0377, *P* = 0.0057) were positively and significantly with SF-36 scale scores on mental health (Figures 2D–F). For physical health evaluated with SF-36 scale scores, it is only significant the relationship with SSRS scores on Subjective Support (r^2^ = 0.0280, *P* = 0.0176) rather than the other two dimensions (Figures 2G–I).

### Association of loneliness and HRQoL in PLWHIV

**Figure 3:**
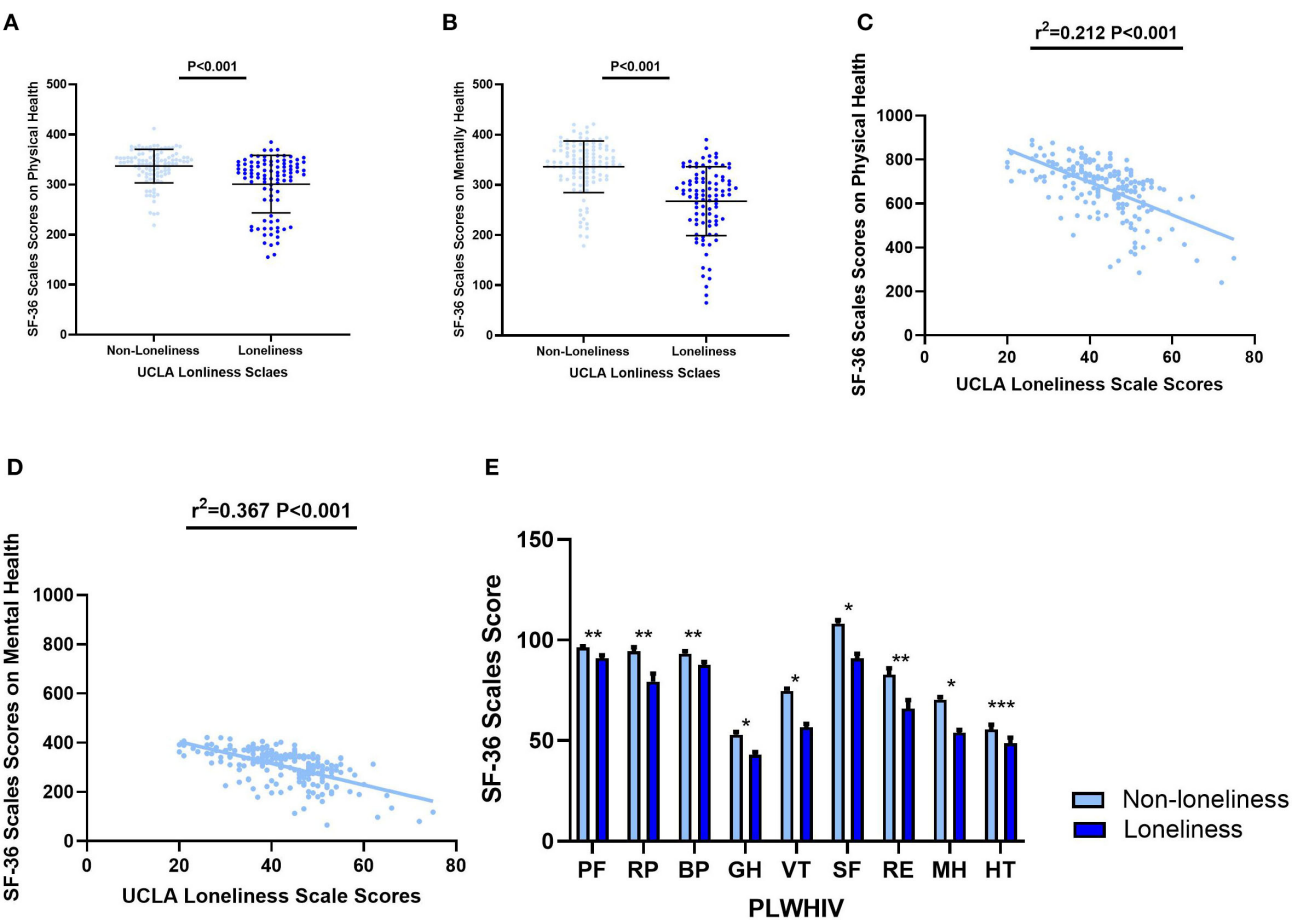
Aspects of HRQoL in the loneliness and non–loneliness groups. **(A)** The SF−36 Scale score on physical health of PLWHIV with loneliness were 336.85 ± 33.52, significantly higher than that without loneliness 300.70 ± 57.36 (*P* < 0.001). **(B)** The SF−36 Scale score on mental health of PLWHIV with loneliness were 335.95 ± 51.38, significantly higher than that without loneliness 267.44 ± 68.69 (*P* < 0.001). **(C)** The SF−36 Scale score on physical health was significantly and negatively co–related with UCLA Loneliness Scale scores. **(D)** The SF−36 Scale score on mental health was significantly and negatively co–related with UCLA Loneliness Scale scores. **(E)** Eight aspects of SF−36 Scale scores in two groups. *P < 0.001; **P < 0.05; ***P > 0.05. Non–loneliness group vs. non–loneliness group: Mean ± SD. PF: 96.31 ± 6.67 vs. 90.96 ± 14.26; RP: 94.39 ± 20.98 vs. 79.26 ± 39.93; BP: 93.23 ± 11.95 vs. 87.52 ± 11.31; GH: 52.91 ± 14.64 vs. 42.96 ± 12.97; VT: 74.63 ± 12.32 vs. 56.70 ± 15.96; SF: 108.18 ± 18.34 vs. 90.82 ± 22.98; RE: 82.87 ± 31.51 vs. 65.96 ± 41.47; MH: 70.28 ± 14.90 vs. 53.96 ± 13.72; HT: 55.53 ± 25.11 vs. 48.67 ± 27.28. SF−36, 36–Item Short Form Survey Instrument; UCLA, The University of California, Los Angeles; PF, Physical Function; RP, Role Physical; BP, Bodily Pain; GH, General Health; VT, Vitality; SF, Social Functioning; RE, Role Emotional; MH, Mental Health; HT, Reported Health Transition.

We next evaluated HRQoL among PLWHIV in the loneliness and non-loneliness groups. The non-loneliness group had higher SF-36 scores in both physical health (336.85 ± 33.52 vs. 300.70 ± 57.36, *P* < 0.001) and mental health (335.95 ± 51.38 vs. 267.44 ± 68.69, *P* < 0.001), as shown in (Figures 3A, B). Levels of HRQoL were significantly and negatively associated with the level of loneliness both in physical health (r^2^ = 0.212, *P* < 0.001) and mental health (r^2^ = 0.367, *P* < 0.001) (Figures 3C, D).

Physical aspects of HRQoL, including physical functioning, physical role functioning, bodily pain, and general health perceptions, were found significantly lower in PLWHIV with loneliness, and the psychological aspects of HRQoL, including vitality, social role functioning, emotional role functioning, also had the same relative relationships (Figure 3E).

### Mediational mechanism of loneliness

**Figure 4:**
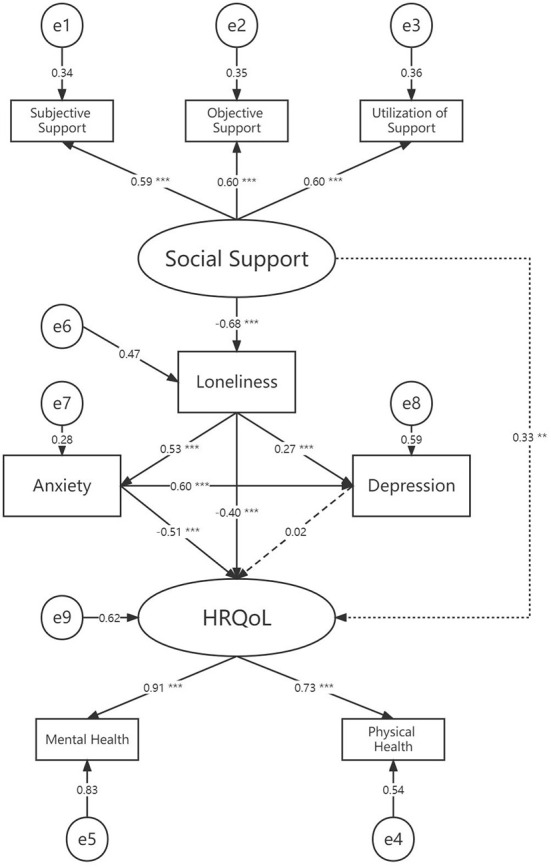
Structural equation model for the mediation mechanism of loneliness with standardized beta weights and significant level. ***P < 0.001; **P < 0.01; Fit statistics: CMIN:20.721; DF:16; CMIN/DF: 1.295; GFI: 0.975; NFI: 0.969; IFI: 0.993; TLI: 0.987; CFI: 0.993; RMSEA: 0.038; HOELTER: 254. HRQoL, Health Related Quality of Life.

We established a conceptual psychosocial model and confirmed it by Structural Equation Model (SEM) (Figure 4). The perceived social support was negatively associated with loneliness (β = −0.68, *P* < 0.001). Loneliness was associated with both anxiety (β = 0.53, *P* < 0.001) and depression (β = 0.27, *P* < 0.001). Together with perceived social support (β = 0.33, *P* < 0.01), both loneliness (β = −0.40, *P* < 0.001) and anxiety (β = −0.51, *P* < 0.001) were predictable for HRQoL. Moreover, anxiety was related to depression (β = 0.60, *P* < 0.001) although depression is not a direct predictor for anxiety (β = 0.05, *P* = 0.846). The model reflected a good model fit: (χ2: 20.721; DF:16; χ2/DF: 1.295; GFI: 0.975; NFI: 0.969; IFI: 0.993; TLI: 0.987; CFI: 0.993; RMSEA: 0.038). The HOELTER was 254 which indicated an adequate sample size.

## Discussion

In our study, we performed a cross-sectional study design with PLWHIV consecutively enrolled to manage psychological measurement and to collect information. The PLWHIV were divided into two groups based on their UCLA Loneliness Scale scores. We found the levels of anxiety and depression were significantly different in the two groups. Moreover, there was a correlation observed between perceived social support and HRQoL of PLWHIV. Furthermore, an association between loneliness and HRQoL of PLWHIV was also observed. Based on the findings above, we proposed a hypothesis model where loneliness played a mediation role between social support and HRQoL, which was confirmed by a method of structural equation model in the present study.

We found the incidence of loneliness is 47% in PLWHIV enrolled in our study. This is much higher than that in normal populations, ranging from 9.2% in Southeast Asia to 14.4% in the Eastern Mediterranean region, as reported before (33). For PLWHIV, studies reported that the incidence of loneliness was 58% in the older population in the USA (15) and 27.97% in adults of all ages in China (18). The prevalence of loneliness was 35.5% in PLWHIV who are men who have sex with men (MSM) (34). Our study observed that PLWHIV have a higher incidence of loneliness than the normal population, which was consistent with the result reported by a previous study (35). Moreover, we further observed that being married is an independent factor associated with loneliness. Together with our study, these findings were not beyond daily life experience, especially in the background of COVID-19 pandemic. People are cut off from daily social contact and the deteriorating economy leads to increasing housing and medical burdens (36).

All those findings indicated a potential relationship between PLWHIV's received care and support from society and their quality of life. Thus, we assessed the association between perceived social support and HRQoL along with its two dimensions. A negative correlation between levels of social support and HRQoL was determined. The impact of social support on mental health was especially more pronounced. The results were consistent with a previous study evaluating Chinese PLWHIV (37). Moreover, our study confirmed that loneliness was positively correlated with depression and anxiety. Patients evaluated with loneliness had higher scores on the SDS and SAS than the others, which was consistent with former studies in general populations (38–40). Similar results were previously validated in elderly PLWHIV (15). It was reported that depression in PLWHIV could lead to severe clinical outcomes, including increased mortality and missing scheduled appointments, which may worsen the management of HIV infection (41). Besides, depression and anxiety may decrease ART adherence, as previously reported (42).

We evaluated whether there was a difference in HRQoL between the loneliness and non-loneliness groups. The results showed that there was a negative correlation between loneliness and HRQoL levels. The higher the levels of loneliness, the worse the physical and mental quality of life. The non-loneliness group had better performance than the loneliness group in physical functioning, role physical, bodily pain, general health, vitality, social functioning, role emotional, and mental health. A study (15) reported that loneliness among older adults living with HIV was associated with poor HRQoL. Another study (43) confirmed similar results in an older population living with HIV. Our study revealed a more comprehensive and extensive relationship between loneliness and HRQoL in a younger PLWHV population. We found that loneliness could affect nearly every aspect of HRQoL among PLWHIV.

Based on our study, we proposed a hypothesis that loneliness may play a mediation role between perceived social support and poor HRQoL among PLWHIV. A structural equation model was adopted to describe the correlation between psychological variables. We found there was a direct and indirect influence of social support on HRQoL where loneliness acted mediately. A high level of social support predicts a low level of loneliness, which predicts a poor HRQoL in turn. In addition, the findings of the associations between loneliness, anxiety, and depression were also included in the model. However, in our model, together with loneliness, anxiety also predicts a poor level of HRQoL, in which depression did not take a mediating role. This is different from some formerly published studies emphasizing the role of depression (44, 45). It is not definite whether loneliness is more prominent and severe among the PLWHIV in the context of the COVID-19 pandemic that led to this difference, which needs more research to determine.

The reasons for the development of loneliness varied for the young and elderly populations in China. In our study, we focused on the impact of social support on the loneliness of a young population with an average age of 27 years. For children infected with HIV, the factors associated with their loneliness may be stigma, discrimination, and a lack of social support (46). However, in older PLWHIV, the factors associated with loneliness were limited support networks and substance use (15). Combining these results, social support or support networks played a vital role that cannot be ignored. Multivariate analysis indicated, in our study, being married was a protective factor for loneliness. The relationship between being married and loneliness was not reported in PLWHIV before. It is unknown whether being married has a protective effect on loneliness in the USA and the European regions where gay marriage is legal, and this is worth more research. There may be many reasons for this clinical situation. It is studied previously in general population that being married was an important way to access intimate companionship and emotional experiences, which protected individuals from loneliness (47). In older general populations, when family members, friends, and neighbors are lost to death and geographic relocation, marital partners become increasingly important in maintaining a sense of social connectedness (48–50). This is where the importance of social support comes to the forefront and validates the views of a former study (15). We believe the same may hold true for PLWHIV.

Studies have reported that the number of Chinese patients infected with HIV is increasing, especially among young people (51). All patients enrolled in our study were Chinese. One rational speculation is that Chinese people were more likely to place greater importance on stable and healthy family relationships influenced by traditional Chinese culture. However, AIDS is a disease that may bring stigma and discrimination to patients, occurs, the patients become victims of social pressure at the same time. A previous study showed that Chinese PLWHIV lack support from their family, friends, and intimate partners (52). Thus, when considering about improvement of loneliness-mediated HRQoL among PLWHIV, interventions on social support may be an intervention worth considering.

## Conclusion

In our study, the prevalence of loneliness was 47% in this population of PLWHIV. Being married is an independent protective factor associated with loneliness in PLWHIV. There was a negative direct and indirect impact of perceived social support on HRQoL where loneliness played a mediation role in this SEM. Besides, PLWHIV with loneliness also had higher levels of anxiety and depression. In the model, together with loneliness, anxiety can predict poor HRQoL. Interventions focused on reducing loneliness or just improving social support for PLWHIV simultaneously may be effective in improving HRQoL among the population.

## Limitations

There are some limitations in our study. Its cross-sectional design precludes inferences about causality and limitations exist with interpreting the findings. Although the sample size was sufficient to complete this study by statistical tests, we expect a larger sample size to maintain the stability of the study results. Next, a longitudinal cohort study to further determine the causality of social support and loneliness on HRQoL needs to be performed.

## Data availability statement

The original contributions presented in the study are included in the article/Supplementary material, further inquiries can be directed to the corresponding author/s.

## Ethics statement

The studies involving human participants were reviewed and approved by Ethics Committee of Nanfang Hospital, Southern Medical University. The patients/participants provided their written informed consent to participate in this study.

## Author contributions

ZQ finished the data analysis and drafted the manuscript. BL finished data collection. GL and HC censored the process. JH, TY, and XX assisted data analyzing. SC and JP designed the study. All authors contributed to the article and approved the submitted version.
