# Supplementary material for: Loneliness as a mediation from social support leading to a decrease of health-related quality of life among PLWHIV
Source: Front Public Health. 2023 Jan 4;10:1067870. doi: 10.3389/fpubh.2022.1067870 (PMC9846772; doi:10.3389/fpubh.2022.1067870)
Supplement: Supplementary file 1 [file Table_1.docx]

**Supplementary Table 1 Proportion reported depression of different levels**

|  | Loneliness Group | Non-Loneliness Group |
| --- | --- | --- |
| No depression 20-44 | 11（11.70%） | 54（50.47%） |
| Mild depression 45-59 | 39（41.49%） | 38（35.51%） |
| Moderate depression 60-69 | 37（39.36%） | 14（13.08%） |
| Severe depression 70-80 | 7（7.45%） | 1（0.94%） |
| Total | 94 | 107 |

Chi square test: P<0.001
