# Supplementary material for: Loneliness as a mediation from social support leading to a decrease of health-related quality of life among PLWHIV
Source: Front Public Health. 2023 Jan 4;10:1067870. doi: 10.3389/fpubh.2022.1067870 (PMC9846772; doi:10.3389/fpubh.2022.1067870)
Supplement: Supplementary file 2 [file Table_2.docx]

**Supplementary Table 2 Proportion reported anxiety of different levels**

|  | Loneliness Group | Non-Loneliness Group |
| --- | --- | --- |
| No anxiety 20-49 | 45（47.87%） | 92（85.98%） |
| Mild anxiety 50-59 | 35（37.23%） | 13（12.15%） |
| Moderate anxiety 60-69 | 10（10.64%） | 2（1.87%） |
| Severe anxiety 70-80 | 4（4.26%） | 0（0%） |
| Total | 94 | 107 |

Chi square test: P<0.001
